# Dual role of transient receptor potential ankyrin 1 in respiratory and gastrointestinal physiology: From molecular mechanisms to therapeutic targets

**DOI:** 10.3389/fphys.2024.1413902

**Published:** 2024-07-03

**Authors:** Kavya Reddy Tekulapally, Ji Yeon Lee, Dong Seop Kim, Md. Mahbubur Rahman, Chul-Kyu Park, Yong Ho Kim

**Affiliations:** ^1^ Gachon Pain Center and Department of Physiology, Gachon University College of Medicine, Incheon, Republic of Korea; ^2^ Department of Anesthesiology and Pain Medicine, Gachon University, Gil Medical Center, Incheon, Republic of Korea

**Keywords:** TRPA1, respiratory system, gastrointestinal system, epithelial cells, immune cells

## Abstract

The transient receptor potential ankyrin 1 (TRPA1) channel plays a pivotal role in the respiratory and gastrointestinal tracts. Within the respiratory system, TRPA1 exhibits diverse distribution patterns across key cell types, including epithelial cells, sensory nerves, and immune cells. Its activation serves as a frontline sensor for inhaled irritants, triggering immediate protective responses, and influencing airway integrity. Furthermore, TRPA1 has been implicated in airway tissue injury, inflammation, and the transition of fibroblasts, thereby posing challenges in conditions, such as severe asthma and fibrosis. In sensory nerves, TRPA1 contributes to nociception, the cough reflex, and bronchoconstriction, highlighting its role in both immediate defense mechanisms and long-term respiratory reflex arcs. In immune cells, TRPA1 may modulate the release of pro-inflammatory mediators, shaping the overall inflammatory landscape. In the gastrointestinal tract, the dynamic expression of TRPA1 in enteric neurons, epithelial cells, and immune cells underscores its multifaceted involvement. It plays a crucial role in gut motility, visceral pain perception, and mucosal defense mechanisms. Dysregulation of TRPA1 in both tracts is associated with various disorders such as asthma, Chronic Obstructive Pulmonary Disease, Irritable Bowel Syndrome, and Inflammatory Bowel Disease. This review emphasizes the potential of TRPA1 as a therapeutic target and discusses the efficacy of TRPA1 antagonists in preclinical studies and their promise for addressing respiratory and gastrointestinal conditions. Understanding the intricate interactions and cross-talk of TRPA1 across different cell types provides insight into its versatile role in maintaining homeostasis in vital physiological systems, offering a foundation for targeted therapeutic interventions.

## 1 Introduction

Transient receptor potential (TRP) channels are sensory receptors involved in detecting various stimuli, ranging from temperature to chemical compounds (Muller et al., 2018; Ujisawa et al., 2024). Among them, Transient Receptor Potential Ankyrin 1 (TRPA1) holds particular interest owing to its responsiveness to numerous environmental irritants and involvement in pain perception and inflammatory processes (Meseguer et al., 2014; Kadkova et al., 2017). TRPA1 is expressed in the nervous system, where it contributes to nociception, itching, and neurogenic inflammation (Nummenmaa et al., 2020; Cerqueira et al., 2023; De Logu et al., 2023), and in the cardiovascular system, where it plays a role in vascular tone regulation (Alvarado et al., 2021). However, the respiratory and gastrointestinal systems are particularly significant owing to their direct contact with environmental stimuli and their susceptibility to TRPA1-mediated responses (Kaji et al., 2012; Kuwaki and Takahashi, 2023) which is comparatively less focused. The intricate orchestration of physiological responses within the gastrointestinal and respiratory tracts depends on the dynamic involvement of the TRPA1 (Yang et al., 2016; Wang M. et al., 2019; Li C. et al., 2023; Liang et al., 2023).

In the respiratory system, TRPA1 has been identified as a sentinel molecular orchestrator that is strategically positioned in the epithelial cells (Wang M. et al., 2019; Luostarinen et al., 2021), sensory nerves (Geppetti et al., 2010; Grace and Belvisi, 2011), and immune cells (Li et al., 2020; Muthumalage and Rahman, 2023) lining the airways. Its pronounced presence in the airway epithelium positions TRPA1 as a frontline sensor for inhaled irritants, triggering immediate protective responses and reflexes aimed at preserving airway integrity (Conklin, 2016). Beyond immediate defense mechanisms, the involvement of TRPA1 in fibroblast-myofibroblast transition and mediation of airway tissue injury underscores its significance in conditions such as severe asthma and fibrosis (Yang and Li, 2016; Li et al., 2020; Yap et al., 2021; Li et al., 2022). TRPA1 is involved in sensory nerve fibers and its activation contributes to nociception, cough reflex, and bronchoconstriction, highlighting its role in both immediate defense and long-term regulatory processes (Grace and Belvisi, 2011; Al-Shamlan and El-Hashim, 2019; Jentsch Matias de Oliveira et al., 2020). Immune cells within the respiratory microenvironment also express TRPA1, suggesting contribution to immune responses and inflammatory processes (Luostarinen et al., 2023; Muthumalage and Rahman, 2023).

When transitioning to the gastrointestinal tract, TRPA1 is equally dynamic and spans epithelial (Wu et al., 2017; Stanzani et al., 2020) and immune cells (Benguettat et al., 2018). Its presence in epithelial cells contributes to the sensing of dietary components, thereby influencing the mucosal defense mechanisms (Kaji et al., 2012), nutrient absorption (Arends, 2018), and barrier function (Maglie et al., 2021).

In this review article, we delve into the nuanced distribution, localization, and physiological functions of TRPA1 in the respiratory and gastrointestinal tracts. By revealing the cellular symphony orchestrated by TRPA1 across different cell types, the review lays the foundation for understanding its pivotal role in maintaining homeostasis in crucial physiological systems. The insights provided herein pave the way for targeted therapeutic interventions in respiratory and gastrointestinal conditions where TRPA1 dysregulation plays a central role.

## 2 General characteristics of TRPA1

### 2.1 Historical perspective and discovery

TRPA1 was initially identified in 1999 when David Julius’s lab at the University of California, San Francisco, investigated the molecular basis of cold and chemical sensitivity in sensory neurons (Bautista, 2015). They were able to clone and characterize TRPA1 as a novel member of the TRP channel family (Meents et al., 2019). This discovery marked a significant step towards understanding the molecular basis of chemo-sensation and nociception.

### 2.2 Discovery of TRPA1 function

TRPA1 is highly sensitive to various chemical irritants, including mustard oil, garlic, and wasabi (Bautista et al., 2005). The key role played by TRPA1 in detecting and responding to noxious chemicals has been identified (Kang et al., 2010; Saito et al., 2012). Studies involving TRPA1 knockout mice have demonstrated that TRPA1 channels are critical for the perception of cold and chemical pain (Reese et al., 2020). TRPA1 expressing sensory neurons were found to be essential for mediating pain responses to a wide range of irritants and inflammatory mediators (Koivisto et al., 2014; Logashina et al., 2019). TRPA1 is also activated by cold temperatures, particularly in the noxious-cold range. This temperature sensitivity contributes to its role in thermosensation (Fajardo et al., 2008; Akashi, 2021). Over the years, studies have linked TRPA1 to various physiological and pathological processes, including neurogenic inflammation, respiratory diseases, and chronic pain conditions (Wei Y. et al., 2022). This has led to investigations into potential therapeutic targeting of TRPA1 for pain management and other disorders.

### 2.3 Domain architecture, including ankyrin repeats and transmembrane regions of TRPA1

**FIGURE 1:**
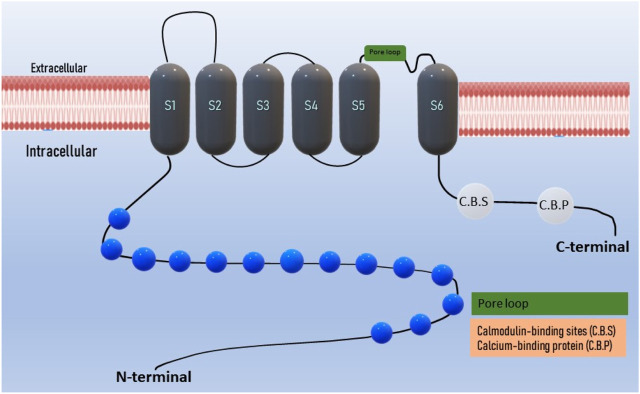
Domain structure of TRPA1 ion channel: The TRPA1 channel has a structural composition comprising six transmembrane domains, with intracellular N- and C- terminals. The transmembrane S5-S6 forms the pore loop. Ankyrin repeats are present in the terminal regions, and the calcium-binding region is located within both the N and C-terminals.

The domain architecture of TRPA1 includes several functional domains and motifs, including ankyrin repeats and transmembrane regions. These domains are crucial for their function as ion channels. TRPA1 contains a series of 14–18 ankyrin repeats located in the N-terminal region of the protein (Gaudet, 2008; Hu et al., 2021). Ankyrin repeats are structural motifs known for their roles in protein-protein interactions (Nilius et al., 2011). In TRPA1, these repeats are involved in protein folding and interaction with various binding partners. The transmembrane regions are responsible for anchoring TRPA1 in the cell membrane and forming the ion channel pore (Nassini et al., 2014; Wang Z. et al., 2019). TRPA1 has six transmembrane segments (S1–S6), intracellular N- and C- terminals, and a pore loop between S5 and S6 (Figure 1) (Story et al., 2003; Chernov-Rogan et al., 2019). TRPA1 possesses a nucleotide-binding domain such as ATP, which is involved in sensing cellular changes at the nucleotide level. This domain may play a role in regulating channel activity (Egbuniwe et al., 2014; Wei Y. et al., 2022; Gawalska et al., 2022). Coiled-coil domains are regions of TRPA1 that contain coiled-coil motifs, which are known for their role in protein-protein interactions and protein oligomerization (formation of multimeric complexes) (Martinez and Gordon, 2019). These domains are involved in the assembly and regulation of the TRPA1 channels. TRPA1 contains multiple calmodulin-binding sites in its C-terminal region (Hasan et al., 2017). Calmodulin is a calcium-binding protein that can modulate TRPA1 activity in response to changes in intracellular calcium levels. AnkTM is a specialized domain found in the N-terminal region of TRPA1 that combines both ankyrin repeats and transmembrane segments (Zayats et al., 2013). It is thought to be involved in the gating mechanism of the channel, and may play a role in channel activation. The exact number and arrangement of ankyrin repeats and transmembrane regions vary between species and TRPA1 isoforms (Jaquemar et al., 1999; Yao et al., 2023). These structural elements collectively contribute to the function of TRPA1 as an ion channel that senses a wide range of chemical and physical stimuli, including irritants, temperature changes, and mechanical forces (Nilius et al., 2011; Moccia and Montagna, 2023). The complex domain architecture of TRPA1 allows it to integrate multiple sensory inputs and respond to various environmental cues (Cordero-Morales et al., 2011; Landini et al., 2022).

### 2.4 Structural insights from X-ray crystallography of TRPA1

The size, membrane-spanning regions, and flexibility of this protein significantly complicate the determination of its structure. Scientists have made progress in understanding the structure of TRPA1 by solving its partial structures using other structural biology techniques. Crystallography and cryo-electron microscopy (cryo-EM) studies have provided insights into the N-terminal region of TRPA1, including the ankyrin repeat domain (Zayats et al., 2013; Paulsen et al., 2015). These studies have shown that the N-terminal domain forms a spiral-shaped structure and that ankyrin repeats may be involved in protein-protein interactions and channel gating (Berrout et al., 2017; Zimova et al., 2018). Determining the transmembrane domain structure of TRPA1 is challenging. Cryo-EM studies have helped identify certain transmembrane segments, providing a general review of the membrane-spanning regions of the channel (Madej and Ziegler, 2018). These studies suggest the presence of multiple transmembrane segments arranged in a manner similar to that of other TRP channels. Certain studies have explored the binding sites of ligands and activators, such as chemical irritants (Noroes et al., 2019). Cryo-EM studies have provided insights into the binding of compounds such as menthol and cinnamaldehyde to specific sites within the TRPA1 channel (Fine and Li, 2021). These binding sites are located in the transmembrane domain, and are important for channel activation.

## 3 Activation mechanisms

### 3.1 TRPA1 activation

**FIGURE 2:**
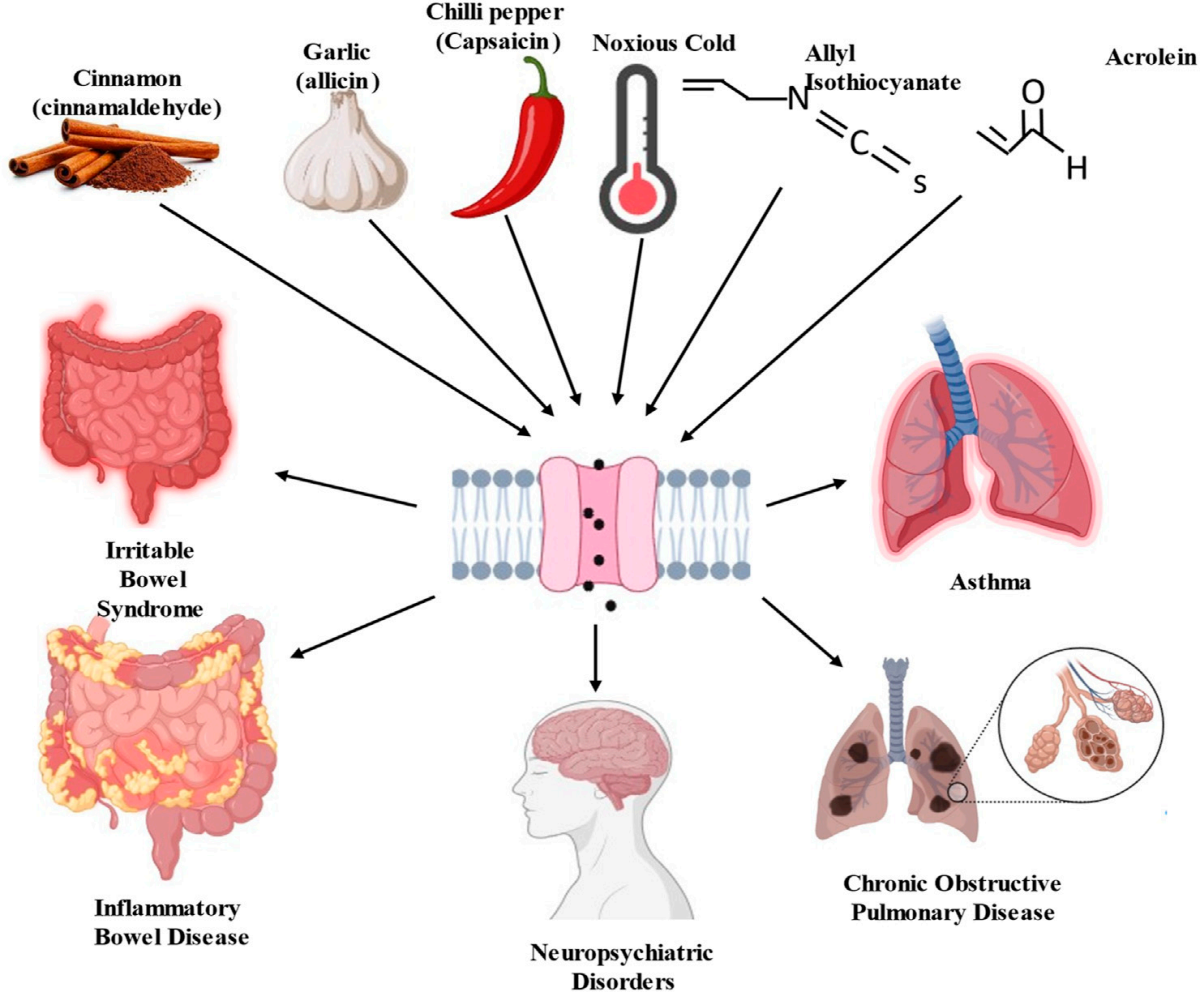
Schematic diagram of transient receptor potential ankyrin 1 (TRPA1) channel activators and consequence of pathological disorders.

TRPA1 channels can be activated by a wide range of stimuli, including chemical irritants, temperature changes, and mechanical forces (Wang Z. et al., 2019; Logashina et al., 2019; Naert et al., 2021). TRPA1 activation is a complex process involving multiple mechanisms, that play crucial roles in sensory perception and nociception (pain perception) (Bandell et al., 2004; Yao et al., 2023). TRPA1 is particularly sensitive to electrophilic chemicals containing electron-deficient atoms or functional groups that can react with nucleophilic residues within channel proteins. Examples of electrophilic activators include allyl isothiocyanate (found in mustard oil), cinnamaldehyde (found in cinnamon), and acrolein (a component of tobacco smoke) (Bandell et al., 2004; Bautista et al., 2005) (Figure 2). Certain reactive oxygen species, such as hydrogen peroxide (H_2_O_2_), can activate TRPA1 by oxidizing specific cysteine residues in the channel (Miyake et al., 2017). Oxidative modification leads to channel opening and ion influx. Certain lipid signaling molecules such as 4-hydroxynonenal (4-HNE) activate TRPA1 (Trevisani et al., 2007). Endogenous activators are produced in response to cellular stress and inflammation. TRPA1 is sensitive to temperature changes, particularly cold temperatures. When exposed to temperatures in the noxious cold range (below approximately 17°C–19 °C), TRPA1 channels can open, allowing for the influx of calcium ions (Ca^2+^) and sodium ions (Na^+^) (Dhaka et al., 2009; Akashi, 2021). This temperature sensitivity contributes to the sensation of cold-induced pain (Caspani and Heppenstall, 2009). TRPA1 can interact with other proteins or signaling molecules, and these interactions can influence its activity. For example, calmodulin, a calcium-binding protein, can bind to TRPA1 and modulate its function in response to changes in intracellular calcium levels (Hasan et al., 2017; Cortes-Montero et al., 2020). Other cellular signaling pathways and modulators such as phosphorylation by protein kinases can also influence TRPA1 activity. The phosphorylation of specific residues within a channel can either enhance or inhibit its function (Schmidt et al., 2009). Notably, TRPA1 channels are expressed in sensory neurons and other cell types throughout the body, allowing them to detect and respond to various environmental cues and physiological changes. The activation of TRPA1 can lead to the generation of electrical signals (action potentials) in sensory neurons, which are then transmitted to the central nervous system, ultimately resulting in the perception of pain or other sensory experiences.

### 3.2 Chemical agonists and physical stimuli of TRPA1

Allyl Isothiocyanate (AITC), which is found in mustard oil and wasabi, is one of the most well-known and potent agonists of TRPA1 (Bandell et al., 2004; Sandor et al., 2016). It is responsible for the pungent and spicy sensations associated with these foods. Hydrogen sulfide (H_2_S), which activates TRPA1, is linked to compounds such as AITC and allicin derivates, which act as H_2_S donors (Andersson et al., 2012; Chung et al., 2020; Li J. et al., 2023). Cinnamaldehyde is found in cinnamon and is known for its sweet and spicy flavor (Bandell et al., 2004). It can activate TRPA1 and contribute to the sensations of heat and spiciness. Although capsaicin is primarily known to activate Transient Receptor Potential Vanilloid 1 channels, it can also activate TRPA1 (Shintaku et al., 2012). Capsaicin, found in chilled peppers, produces a burning sensation. 4-HNE is a lipid-derived aldehyde produced during oxidative stress, which activates TRPA1. It is also an endogenous TRPA1 agonist (Trevisani et al., 2007). Formaldehyde is a strong irritant that activates TRPA1. Mice deficient in TRPA1 show a notable reduction in pain responses elicited by formaldehyde (Macpherson et al., 2007). It is used in various industrial applications and can be found in household products. The health consequences of inhaling acrolein, a significant electrophile in fires, tobacco smoke, and vehicle exhaust, indicate the pivotal role of TRPA1 (Conklin et al., 2017). Upon interaction with TRPA1, acrolein induces the release of proinflammatory neuropeptides, such as CGRP and substance P, from sensory nerve endings in the lungs, trachea, and larynx (Achanta and Jordt, 2017). Nitro oleic acid, an electrophilic fatty acid nitroalkene generated from redox reactions, has been shown to activate TRPA1 and TRPV1 in primary dorsal root ganglion neurons sourced from adult male rats. It appears to induce desensitization of their responses to TRPA1 and TRPV1 agonists. This suggests that nitro-oleic acid could have clinical utility in reducing neurogenic inflammation and specific types of painful sensations by modulating TRPA1 expressing nociceptive afferents (Sculptoreanu et al., 2010; Zhang et al., 2014). ROS and other oxidative compounds activate TRPA1 by oxidizing specific cysteine residues within the channel. Inflammatory mediators released during tissue injury or inflammation can sensitize TRPA1 and reduce its activation threshold (Takahashi and Ohta, 2013). This sensitization can lead to increased TRPA1 responsiveness to other agonists.

TRPA1 is triggered by physical stimuli such as extremely cold temperatures below 17°C, heat stress, mechanical stretch, osmolarity, ultraviolet ray, and photosensitizing agent (Hill and Schaefer, 2009; Raisinghani et al., 2011; Moparthi et al., 2016). TRPA1 activation can occur in response to cell swelling induced by hypotonic conditions, where cells experience lower osmotic pressure (Fujita et al., 2018; Stinson et al., 2023). TRPA1 channel in the skin is involved in both prolonged and painful mechanical stimulus-induced postoperative pain, while the spinal TRPA1 channel primarily contributes to postoperative pain caused by non-painful mechanical stimuli (Wei et al., 2012). TRPA1 is sensitive to cold temperatures. It can be activated by temperatures in the noxious cold range, typically below approximately 17°C–19 °C (Story et al., 2003; Dhaka et al., 2009; Akashi, 2021). TRPA1 is the primary mediator of cold-evoked responses in vagal visceral neurons, underscoring its critical role in visceral thermosensation (Fajardo et al., 2008). Mechanical stimuli such as pressure or membrane stretching can activate TRPA1. This activation mechanism is thought to be involved in the sensation of mechanical pain or hypersensitivity (Wei H. et al., 2022). Alterations in the membrane voltage modulate TRPA1 activity. Voltage-dependent gating can influence TRPA1 channel function (Shen et al., 2020). Changes in pH, particularly under acidic conditions, can affect TRPA1 activity (Dhaka et al., 2009). Low pH sensitizes TRPA1 to other activators. The sensitivity of TRPA1 to these stimuli allows it to function as a multimodal receptor that detects various noxious or irritating conditions in the body. Activation of TRPA1 can lead to the perception of pain, tingling, burning, and other sensory experiences, making it an important player in sensory physiology and nociception (Morgan et al., 2009; Maglie et al., 2021).

## 4 TRPA1 in the respiratory system

### 4.1 Localization and function

The respiratory tract, a dynamic interface between the external environment and internal milieu, harbors TRPA1, a molecular orchestrator with diverse distribution and localization patterns across key cell types. In this exploration, we delve into the spatial dynamics of TRPA1 within the respiratory tract, focusing on its presence in epithelial cells (Wang M. et al., 2019), sensory nerves (Geppetti et al., 2010; Grace and Belvisi, 2011; Luostarinen et al., 2021), and immune cells (Li et al., 2020; Muthumalage and Rahman, 2023). TRPA1 is highly expressed in epithelial cells lining the respiratory airways.

Inhaled irritants and environmental challenges trigger TRPA1 (Buch et al., 2013; Kunkler et al., 2014; Rapp et al., 2023). Activation of TRPA1 in epithelial cells triggers immediate protective responses, initiating reflexes aimed at preserving airway integrity (Mukhopadhyay et al., 2011).

TRPA1 is expressed in the airway epithelium, including the lining of the upper and lower respiratory tracts (Mukhopadhyay et al., 2011; Buch et al., 2013). TRPA1 is implicated in the mediation of airway tissue injury and inflammation (Wang M. et al., 2019; Sun et al., 2021).

Additionally, it facilitates fibroblast-myofibroblast transition (FMT), a process that contributes to airway remodeling, posing a significant challenge in conditions such as severe asthma and fibrosis (Wang M. et al., 2019). TRPA1 channels are activated in both primary sensory neurons and heterologous cells when exposed to oxidants such as hypochlorite and H_2_O_2_ (Bessac et al., 2008). These oxidants, which pose a potential threat to airway function and integrity, trigger TRPA1 channels, leading to oxidant-induced respiratory depression, nasal obstruction, sneezing, coughing, and pain (Bessac et al., 2008). Abundant expression of TRPA1 characterizes the sensory nerve fibers innervating the respiratory tract, aligning this molecular entity with an intricate network of respiratory reflexes (Hjerling-Leffler et al., 2007; Kim et al., 2010).

Activation of TRPA1 in sensory nerves contributes to nociception and the perception of pain, and amplifies sensitivity to respiratory irritants (Belvisi et al., 2011). The consequences of TRPA1 activation in sensory nerves are manifested in reflexive responses, such as coughing and bronchoconstriction, revealing its significance in both immediate defense mechanisms and the regulation of long-term respiratory reflex arcs (Deering-Rice et al., 2015). Beyond its role in the epithelial and sensory components, TRPA1 is involved in immune cells residing within the respiratory microenvironment. The expression of TRPA1 in immune cells, including macrophages, and mast cells (Prasad et al., 2008), suggests that it is involved in immune responses and inflammatory processes (Naert et al., 2021). The activation of TRPA1 in these immune cells could potentially modulate mast cell degranulation and the release of proinflammatory mediators, such as histamine, serotonin, and some cytokines (TNF-α), contributing to the regulation of immune responses and the overall inflammatory milieu within the respiratory system. Understanding the spatial nuances of TRPA1 across different cell types reveals its significance in respiratory homeostasis and lays the foundation for targeted therapeutic interventions for respiratory conditions in which TRPA1 dysregulation plays a pivotal role. A summary of TRPA1 agonists and antagonists, along with their involvement, roles, and mechanisms in the respiratory system, is presented in Table 1.

**TABLE 1:** Roles played by TRPA1 in the respiratory system, detailing its cellular involvement and implications for related disorders.

Agonists	Antagonists	Involvement and roles	Mechanisms	References
Hypochlorite, hydrogen peroxide	BI01305834	Frontline sensor for inhaled irritants and environmental challenges	Activation triggers immediate protective responses, initiating reflexes for airway integrity	Bessac et al. (2008), Mukhopadhyay et al. (2011), Buch et al. (2013), Wang et al. (2019a)
Crotonaldehyde, Acrolein	GDC-0334	Mediator of airway tissue injury and inflammation Facilitates fibroblast Myofibroblast transition—Involved in respiratory depression, nasal obstruction, coughing and pain	Activation in sensory nerves contributes to nociception and reflexive responses	Hjerling-Leffler et al. (2007), Simon and Liedtke (2008), Kim et al. (2010), Long et al. (2019)
Bradykinin	-	Modulation of sensory nerve activity in responses to inflammatory mediators Contribution to cough reflex	TRPA1 activation enhances sensitivity to irritants and propagates signals leading to cough reflex	Birrell et al. (2009), Grace and Belvisi (2011), Kanezaki et al. (2012), Mukhopadhyay et al. (2016)
Fine Particulate Matter (PM2.5)	Cannabidiol, N-acylethanolamine	Contribution to airway neurogenic inflammation Release of inflammatory neuropeptides	Channels contribute to airway inflammation and contraction of airway smooth muscles	Xu et al. (2019), Balestrini et al. (2021), van den Berg et al. (2021), Luostarinen et al. (2023)
-	BI01305834	Protection against allergen-induced bronchoconstriction and airway hyperresponsiveness	Inhibition of TRPA1 by BI01305834 prevent AHR, EAR, and LAR *in vivo* and reverses bronchoconstriction	van den Berg et al. (2021)
-	GDC-0334, HC-030031	Orchestrator of responses to environmental irritants and endogenous signals	Regulates cough reflex, bronchoconstriction, and airway inflammation	Lowin et al. (2015), Balestrini et al. (2021), Landini et al. (2022)

### 4.2 Physiological functions

The TRPA1 channel has been identified as a pivotal molecular orchestrator of the respiratory system, deftly mediating responses to a spectrum of environmental irritants and endogenous signals (Zhao et al., 2020). This comprehensive exploration sheds light on the multifaceted role of TRPA1 in orchestrating cough reflex (Grace and Belvisi, 2011; Al-Shamlan and El-Hashim, 2019), bronchoconstriction (Jentsch Matias de Oliveira et al., 2020; van den Berg et al., 2021), and airway inflammation (Li et al., 2022) in the context of external and internal stimuli. Various irritants capable of activating TRPA1 receptors in airway sensory neurons have been identified, leading to neurogenic inflammation and heightened respiratory sensitivity (Balestrini et al., 2021). The recognition of TRPA1 activation by harmful substances present in cigarette smoke and polluted air, including crotonaldehyde, acrolein, and oxidizing agents such as hydrogen peroxide, represents a significant discovery (Simon and Liedtke, 2008). The activation of TRPA1 has been identified in four distinct phenotypes associated with cough hypersensitivity. This discovery underscores the presence of heterogeneity in cough pathways, offering a novel avenue for personalized management of chronic refractory cough (Long et al., 2019). Consequently, it stands out as one of the most promising targets currently recognized for the development of antitussive drugs (Birrell et al., 2009).

The significance of TRPA1 extends beyond environmental irritants to endogenous signaling molecules, particularly inflammatory mediators (Yang et al., 2023). TRPA1 activation contributes to the modulation of sensory nerve activity (Wang et al., 2008). This activation enhances the sensitivity to irritants and propagates signals leading to a cough reflex, thereby amplifying the responsiveness of the respiratory system to both external and internal triggers. When activated by irritants, the TRPA1 channels initiate reflex responses that protect the respiratory system (Mukhopadhyay et al., 2016). TRPA1 activation in the airway sensory nerves can lead to the initiation of the cough reflex (Grace and Belvisi, 2011; Kanezaki et al., 2012), which helps expel irritants and foreign particles from the airways, preventing them from entering the lungs (Geppetti et al., 2010). Changes in TRPA1 expression and function in the sensory neurons may contribute to neuropathic pain in patients with diabetes. TRPA1 has been implicated in joint pain and inflammation associated with rheumatoid arthritis TRPA1 (Luostarinen et al., 2023). Excessive TRPA1 activation or sensitization can lead to increased pain sensitivity and contribute to chronic pain syndromes (De Logu et al., 2020).

TRPA1 activation stimulates mucus production in the airways (Manneck et al., 2021; Kumar et al., 2022). It is another protective mechanism aimed at trapping and removing irritants and pathogens from the respiratory system. A previous study explored the activation of TRPV1 and TRPA1 in response to fine particulate matter (PM2.5) exposure (Xu et al., 2019). These channels contribute to airway neurogenic inflammation, triggering the release of inflammatory neuropeptides (including neurokinin A, substance P) and pro-inflammatory cytokines (TNF-α, and IL-1β). This activation has the potential to initiate early airway inflammation and contraction of airway smooth muscles (Xu et al., 2019). The exact mechanism behind smoke-induced bronchoconstriction is not fully understood; inhaled cigarette smoke directly stimulates sensory nerve endings in the respiratory tract, specifically targeting C fiber endings and rapidly adapting receptors in the airways and lungs (Kou and Lee, 1990; Lee et al., 2018). The mechanism of TRPA1 activation is linked to bronchoconstriction, as the activation of sensory nerve terminals expressing TRPA1 channels can lead to respiratory symptoms such as bronchoconstriction and mucus secretion. When TRPA1 channels, positioned at these nerve terminals, are activated, they contribute to these respiratory symptoms by inducing neurogenic inflammation and exerting direct effects on airway smooth muscle, leading to bronchoconstriction (Grace et al., 2014; van den Berg et al., 2021). The newly developed TRPA1 antagonist BI01305834 effectively prevented ovalbumin-induced bronchoconstriction in guinea pigs. Sensory nerve fibers densely packed in the respiratory epithelium, from the nose to the lower airways, act as a key defense mechanism by detecting irritants and harmful agents. This initiates coordinated reflex responses, including bronchoconstriction, mucus, secretion, sneezing, and coughing (Geppetti et al., 2010). *In vivo*, BI01305834 effectively inhibits airway hyperresponsiveness (AHR) and both the early and late asthmatic reactions (EAR and LAR) (van den Berg et al., 2021). Moreover, in *ex vivo* settings, it hinders allergen- and histamine-induced airway narrowing and reverses allergen-induced bronchoconstriction independent of inflammation. Histamine, a central mediator released from mast cells during allergic reactions, contributes to airway obstruction by inducing smooth muscle contraction, bronchial secretion, and airway mucosal edema. Additionally, it affects various cell types involved in immune and inflammatory responses (Gelfand, 2002; Yamauchi and Ogasawara, 2019). AITC did not induce airway narrowing or histamine release. Therefore, the protective effect of BI01305834 is unlikely to be attributed to histamine release inhibition by the highest concentration antagonist. Consequently, mast cell-mediated effects, notably allergen-induced histamine release, cannot fully elucidate the alleviation of asthma symptoms following TRPA1 antagonism (van den Berg et al., 2021). TRPA1 has emerged as a versatile conductor in respiratory symphonies that orchestrates responses to environmental irritants and endogenous signals. Its involvement in the cough reflex, bronchoconstriction, and airway inflammation underscores its pivotal role in maintaining respiratory homeostasis.

### 4.3 Pathological implications

TRPA1, a prominent “chemosensor,” plays a crucial role in sensory exogenous irritants and endogenous pro-inflammatory mediators (Bodkin and Brain, 2011; Mukhopadhyay et al., 2016; Marsh et al., 2020). Its significance in safeguarding the airway is evident through its involvement in respiratory disorders, such as chronic cough, asthma, chronic obstructive pulmonary disease (COPD), allergic rhinitis, and cystic fibrosis (Mukhopadhyay et al., 2016). TRPA1 activation can also cause airway constriction (Lai et al., 2023). This response is part of a protective mechanism against harmful airborne substances (van den Berg et al., 2021). However, excessive or chronic TRPA1 activation can contribute to airway hyperreactivity and respiratory conditions such as asthma (Jha et al., 2015). In asthma, characterized by recurrent episodes of wheezing, breathlessness, and coughing, the dysfunction of TRPA1 is linked to heightened responsiveness of the airways. Dysregulated TRPA1 signaling may contribute to exaggerated bronchoconstriction, which is a hallmark of asthma attacks (Gawalska et al., 2023). Hypersensitivity of TRPA1 in sensory nerves may amplify the perception of environmental irritants, leading to heightened cough reflex and exacerbation of asthmatic symptoms. The effects of Th1- and Th2-type inflammation on TRPA1 expression and function in A549 human lung epithelial cells were investigated (Luostarinen et al., 2023). These findings revealed the upregulation of TRPA1 expression and function in lung epithelial cells during inflammatory conditions. Specifically, IFN-γ enhanced TRPA1 expression, whereas IL-4 and IL-13 suppressed it in a JAK-STAT6 dependent manner, introducing a novel aspect to the regulation. Additionally, TRPA1 modulates the expression of genes related to innate immunity and lung disease (Luostarinen et al., 2023).

In chronic obstructive pulmonary disease characterized by persistent respiratory symptoms (Landini et al., 2022), TRPA1 dysfunction may contribute to chronic bronchoconstriction and obstructive airway changes. Dysregulated TRPA1 signaling in response to environmental irritants may lead to recurrent exacerbations, triggering an acute worsening of symptoms and hastening disease progression. In a study involving 143 patients with COPD and 104 smokers with post-bronchodilator forced expiratory volume in one second (FEV1)/forced vital capacity (FVC) > 70%, the TRPA1 rs4738202 polymorphism was linked to a predisposition to COPD. These findings suggest that the TRPA1 rs4738202 polymorphism has the potential to serve as a biomarker of COPD susceptibility (Naumov et al., 2021). GDC-0334 is a TRPA1 antagonist with high potency, selectivity, and oral bioavailability. In preclinical studies, its inhibitory effect on TRPA1 function in both airway smooth muscles and sensory neurons resulted in reduced edema, dermal blood flow, coughing, and allergic airway inflammation (van den Berg et al., 2021). These findings offer a therapeutic basis for considering TRPA1 inhibition as a potential clinical intervention for asthma (Balestrini et al., 2021). The regulation of airway inflammation involves TRPA1, and any malfunction in this process may disturb the complicated balance of inflammatory responses, contributing to the onset of chronic obstructive pulmonary disease (COPD) (Landini et al., 2022). Allergic airway inflammation, often observed in conditions such as allergic rhinitis and asthma, may exhibit a heightened sensitivity to TRPA1 dysfunction (Caceres et al., 2009; Balestrini et al., 2021). Dysregulated TRPA1 activation in sensory nerves and immune cells may contribute to sensitization and amplify allergen responses (Caceres et al., 2009; Nassini et al., 2012). This hypersensitivity can manifest as exacerbated bronchoconstriction, heightened cough reflex, and increased inflammatory responses upon exposure to allergens.

**FIGURE 3:**
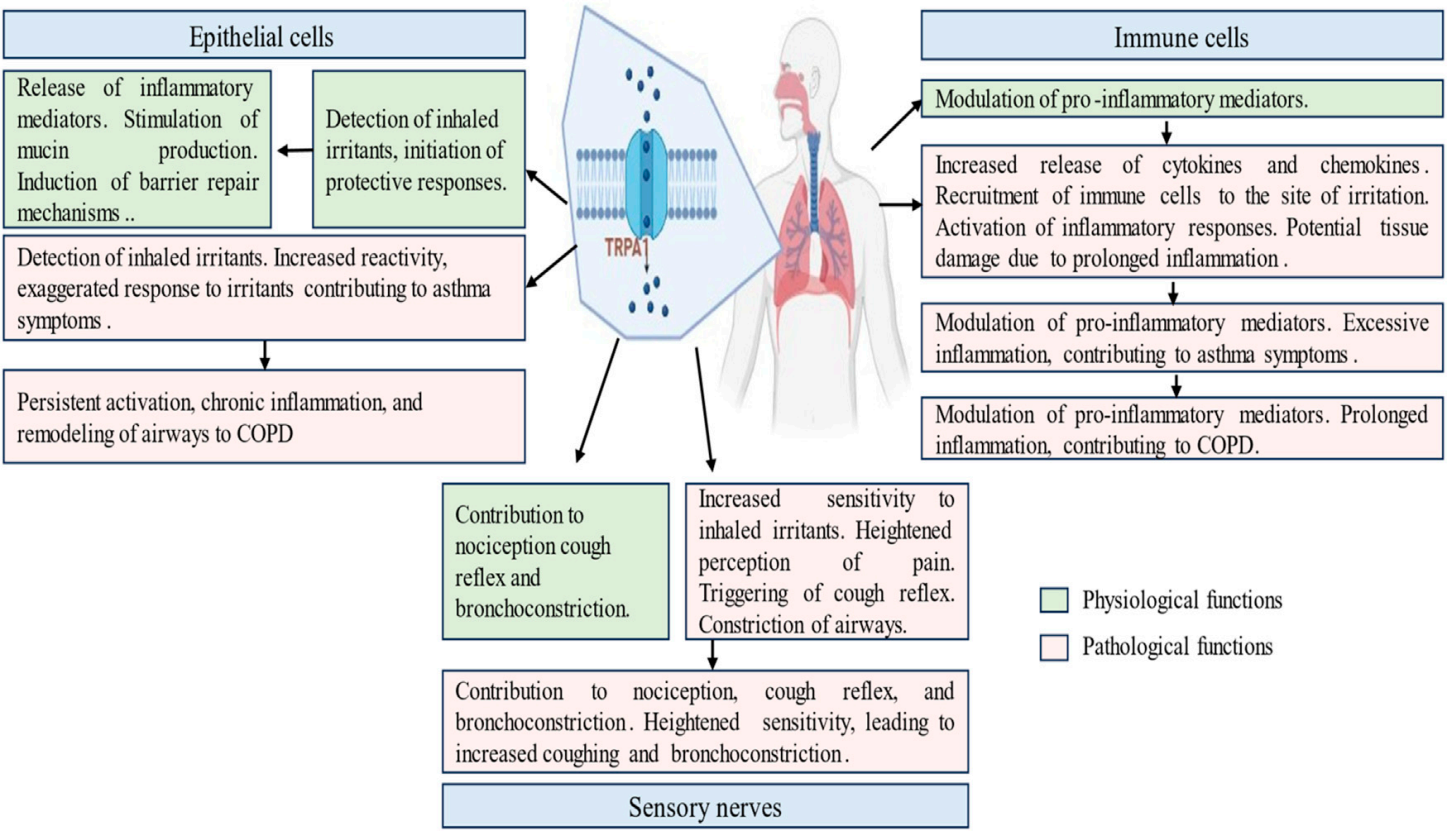
Physiological and pathological roles of transient receptor potential ankyrin 1 (TRPA1) channel in the respiratory diseases. This figure illustrates the multifaceted involvement of TRPA1 in respiratory ailments, including normal physiological and pathways of asthma and COPD induction. It delineates the role played by TRPA1 in exacerbating airway constriction and hypersensitivity, contributing to disease progression. It elucidates TRPA1 modulation by inflammatory mediators and genetic factors, offering insights into disease susceptibility. It highlights the presence of TRPA1 in epithelial cells, sensory nerves and immune cells.

TRPA1 activation in the airways can contribute to airway constriction and hyperreactivity. Excessive TRPA1 activation by environmental irritants may exacerbate asthmatic symptoms and bronchoconstriction. The inhibition of TRPA1 selectively reduces pulmonary inflammation and mitigates airway hyperreactivity in mouse and guinea pig models of asthma (Balestrini et al., 2021). In mice sensitized to ovalbumin, the inhibition of TRPA1 resulted in a reduction in inflammation, mucus production, and airway hyperreactivity, while leaving the immune response triggered by the allergen unaffected (Facchinetti F, 2010). The inhibition of TRPA1 by cannabidiol decreased cell viability, proliferation, and cytokine production, indicating its potential anti-arthritic activity under inflammatory conditions (Landini et al., 2022). In patients with rheumatoid arthritis, N-acylethanolamine anandamide (AEA), a dual inhibitor of fatty acid amide hydrolase/cyclooxygenase-2 (FAAH/COX-2), demonstrated the ability to inhibit MAPK signaling. Furthermore, AEA desensitizes TRPA1, resulting in reduced of interleukin (IL) levels and COX-2-dependent MMP-3 expression (Lowin et al., 2015; Yao et al., 2023). GDC-0334 effectively suppresses TRPA1 function in airway smooth muscle and sensory neurons, leading to reduced edema, dermal blood flow (DBF), cough, and allergic airway inflammation in multiple preclinical species (Balestrini et al., 2021). The potential contribution of TRPA1 dysfunction to respiratory diseases such as asthma, Chronic Obstructive Pulmonary Disease, and allergic airway inflammation is a complex interplay between altered sensory perception, inflammatory dysregulation, and structural changes in the airways. TRPA1 dysregulation and respiratory pathophysiology, offer insights into potential therapeutic interventions for the management of respiratory disorders (Figure 3).

## 5 TRPA1 in the gastrointestinal tract

### 5.1 Expression and localization

The gastrointestinal tract, a complex system that orchestrates digestion and nutrient absorption, harbors a TRPA1 channel with a dynamic expression pattern (Ding et al., 2022). This elaborate exploration revealed a nuanced distribution of TRPA1 in various segments of the gastrointestinal tract, accentuating its presence in enteric neurons (Poole et al., 2011; Someya et al., 2015), epithelial cells (Kono et al., 2013), and immune cells (Benguettat et al., 2018). TRPA1 is expressed in sensory neurons within the gastrointestinal tract, including the stomach and intestine (Doihara et al., 2009; Yu et al., 2016). Their role in the gastrointestinal tract is to detect and respond to various stimuli and changes in the gut environment. A recent study has investigated the involvement of TRPA1 in the regulation of gastrointestinal motility. The activation of TRPA1 results in the release of serotonin from enterochromaffin cells, which influences gut reflexes and motility (Nozawa et al., 2009). Activation of TRPA1 in gut sensory neurons can influence gut reflexes, including peristalsis (the movement of food through the digestive tract) and sensitivity to the mechanical and chemical stimuli (Nozawa et al., 2009; Hassan et al., 2020). Inflammatory mediators such as aldehydes 4-hydroxy-2-hexenal (HHE) and malondialdehyde are released during gut inflammation; increased levels of aldehydes sensitize TRPA1, thereby lowering its activation threshold (Lennertz et al., 2012; Larsson et al., 2016). This can lead to an increased responsiveness of TRPA1 in the gut, contributing to abdominal pain and hypersensitivity in conditions such as irritable bowel syndrome (IBS). Altered TRPA1 function is associated with gastrointestinal disorders such as inflammatory bowel disease (IBD) (Kumar et al., 2022). Dysregulated TRPA1 activity in the gut sensory neurons may contribute to the symptoms experienced by individuals with these conditions.

TRPA1 is integral to the sensitization of esophageal sensory afferents by inflammatory mediators, contributing significantly to esophageal nociception. Mast cell tryptase, acting through protease-activated receptor 2 (PAR2)- mediated pathways, sensitizes sensory nerves and induces hyperalgesia (Yu et al., 2009). TRPA1 activation, facilitated by a PAR2-dependent mechanism, increases TRPA1 sensitivity, leading to mechanical hypersensitivity in esophageal vagal C-fibers (Yu et al., 2009). Immunostaining was used to examine the expression of TRPA1 in small-to-medium sized vagal nodoses and jugular neurons labelled with esophageal dil (Yu et al., 2016). Enterochromaffin cells exhibit high expression of TRPA1, and TRPA1 agonists effectively stimulate enterochromaffin cell functions, including the elevation of intracellular Ca^2+^ levels and the release of 5-HT. Furthermore, AITC induces contraction in isolated guinea pig ileum through activation of the 5-HT3 receptor (Nozawa et al., 2009). TRPA1 extends to epithelial cells in the stomach lining (Camacho et al., 2015). TRPA1 activation may contribute to the detection of irritants and modulation of protective responses, influencing gastric mucus secretion and mucosal integrity. Enteroendocrine cells (EEC) expressing TRPA1 are prevalent in the duodenum and jejunum, less common in the distal small intestine, and entirely absent in the stomach and large intestine (Cho et al., 2014). TRPA1 was observed in EEC co-containing cholecystokinin (CCK) and 5-hydroxytryptamine (5HT), as well as in a subset of cells expressing 5HT without CCK. Notably, TRPA1 is not present in CCK cells lacking 5HT expression or in EEC containing glucagon-like insulinotropic peptides (Cho et al., 2014). TRPA1 is present in primary extrinsic afferent nerves that innervate the esophagus, stomach, intestine, and colon (Yu et al., 2016). 55% of the gastroesophageal vagal afferents exhibited TRPA1 localization. The role of TRPA1 in the gut is becoming clearer; however, the results are still in the preliminary stages (Blackshaw et al., 2010). Nociceptive dorsal root ganglion (DRG) neurons commonly exhibit TRPA1 and TRPV1 co-localization. Activation of TRPA1 agonists induces cross-desensitization in these neurons in response to capsaicin and *vice versa* (Kollarik and Undem, 2004; Blackshaw et al., 2010). Modulation of pacemaker potentials by menthol involves TRPA1 channels (Kim et al., 2016). Upregulation of blood flow in the rat small intestine is facilitated by the epithelial TRPA1-dependent adrenomedullin. In intestinal epithelial cells (IECs), TRPA1 may play a crucial role in the regulation of bowel microcirculation through the release of adrenomedullin (ADM) (Kono et al., 2013). TRPA1 in IECs is implicated in the ADM-mediated vasodilatory effects of daikenchuto (TU-100), a traditional Japanese herbal medicine that increases intestinal blood flow (IBF). TRPA1 antagonists abolish the vasodilatory effects of TU-100 (Kono et al., 2013).

Within the colonic tissue, TRPA1 is primarily expressed in mesenchymal cells of the lamina propria, which is distinctly different from its distribution in the small intestine. These cells co-expressed COX1 and microsomal prostaglandin E synthase-1. Colonic contraction induced by intracolonic administration of TRPA1 agonists was inhibited by a prostaglandin E2 (PGE2) receptor 1 antagonist. TRPA1 activation in cultured human fibroblasts leads to calcium influx and PGE2 release. In animals treated with dextran sulfate sodium, both TRPA1 and its endogenous agonist were markedly increased in the colonic lamina propria, contributing to abnormal colorectal contractions. Pharmacological and genetic inhibition of TRPA1 significantly prevents abnormal colorectal contractions (Yang et al., 2019). TRPA1 extends beyond the neuronal and epithelial domains to immune cells residing in the gastrointestinal tract (Naert et al., 2021). Immune cells such as macrophages and mast cells express TRPA1, suggesting a contribution to local immune responses and inflammatory processes (Chen et al., 2020). TRPA1 plays a dual role in immunity by functioning as a detector of cellular stress, tissue injury, and external noxious stimuli, leading to defensive responses (Naert et al., 2021). However, aberrant regulation contributes to escalation of inflammatory conditions. Future investigations should focus on elucidating the functional properties of TRPA1 in immune cells, which is a crucial step in understanding its involvement in inflammation and exploring its potential as a therapeutic target (Naert et al., 2021). TRPA1 orchestrates a symphony of responses across different segments of the gastrointestinal tract.

### 5.2 Physiological functions

The TRPA1 channel is at the forefront of chemical sensing in the gastrointestinal tract, orchestrating a symphony of responses to a myriad stimuli ranging from dietary components (Fothergill et al., 2016) to inflammatory signals (Cseko et al., 2019). This extensive exploration has delved into the multifaceted physiological functions of TRPA1, accentuating its profound involvement in gut motility (Legrand et al., 2020), visceral pain perception (Pereira et al., 2013; Chen et al., 2019), and the complex defense mechanisms that safeguard mucosal integrity (Ohashi et al., 2023). TRPA1, positioned strategically in epithelial cells throughout the gastrointestinal tract, plays a pivotal role in sensory transduction of dietary components. Its responsiveness to a diverse array of chemicals, including those found in spicy foods (e.g., isothiocyanates) (Wu et al., 2017; Radhakrishnan et al., 2023) and pungent substances (e.g., allicin in garlic) (Sodhi et al., 2021), underscores its significance in translating the chemical landscape of ingested nutrients into cellular signals. Considering its physiological function in the gastrointestinal tract, TRPA1 agonists, such as allicin and AITC, might exert a regulatory influence on gastrointestinal motility (Tsuchiya and Kawamata, 2019). The stimulation of TRPA1 receptors in the intestine by dietary compounds such as AITC, cinnamaldehyde, and linalool (Fothergill et al., 2016; Kashiwadani et al., 2021) triggers mucosal defense mechanisms (Table 2). When food components activate TRPA1 on enterocytes, they enhance transmucosal ion currents, which in turn facilitate nutrient absorption (Fothergill et al., 2016). TRPA1 activation in response to dietary compounds initiates mucosal defense mechanisms. These include the modulation of epithelial barrier function, mucus secretion, and antimicrobial peptide release (Geppetti et al., 2010; Majhi et al., 2021; van den Berg et al., 2021).

**TABLE 2:** Roles played by TRPA1 in the gastrointestinal system, detailing its cellular involvement and implications for related disorders.

Agonists	Antagonists	Involvement and roles	Mechanisms	References
Isothiocyanates, Allicin	Capsazepine	Plays a pivotal role in sensory transduction of dietary components Responds to a diverse array of chemicals	Activation influences mucosal defense mechanisms, including barrier function and mucus secretion	Wu et al. (2017), Tsuchiya and Kawamata (2019), Radhakrishnan et al. (2023)
Allyl Isothiocyanate, Cinnamaldehyde, Linalool	-	Modulates epithelial barrier function, mucus secretion, and antimicrobial peptide release Influences nutrient absorption	Agonists induce mucosal defense mechanisms, enhancing nutrient absorption	Geppetti et al. (2010), Majhi et al. (2021), Luostarinen et al. (2023)
Inflammatory Mediators	HC-0300031	Sensitization of TRPA1 by inflammatory mediators lowers activation threshold, contributing to hypersensitivity	TRPA1 sensitization leads to increased responsiveness and contributes to abdominal pain in IBS	Nozawa et al. (2009), Cho et al. (2014), Kumar et al. (2022)
Mast Cell Tryptase, PAR2	-	Sensitization of esophageal sensory afferents by inflammatory mediators Contributes to esophageal nociception	Activation through PAR2-mediated pathways heightens TRPA1 sensitivity, inducing mechanical hypersensitivity	Yu et al. (2009), Yu et al. (2016)
-	TU-100 (Adrenomedullin)	Regulator of bowel microcirculation through the release of adrenomedullin in intestinal epithelial cells	TRPA1 in IECs plays a role ADM-mediated vasodilatory effect, increasing intestinal blood flow	Kono et al. (2013)
-	Prostaglandin E2 Receptor 1 Antagonist	Involved in abnormal colorectal contractions and inflammation in the colon	TRPA1 activation in colonic mesenchymal cells leads to abnormal colorectal contractions and inflammation	Yang et al. (2019)
Hsp90 Inhibition	Allyl Isothiocyanate (AITC)	Regulates anti-inflammatory impact of Hsp90 inhibition in macrophages during LPS or PMA stimulation	TRPA1 activation enhances Hsp90 inhibition-mediated responses against LPS or PMA stimulation	Radhakrishnan et al. (2023)
-	HC-030031	Contributor to immune modulation within the GI microenvironment	Dysregulated TRPA1 activation in immune cells may perpetuate low-grade inflammation in IBS	Beckers et al. (2021), Naert et al. (2021)

The involvement of TRPA1 in these processes highlights its dual role as a sensor and effector, contributing to the preservation of mucosal integrity in the face of dietary challenges. In the presence of inflammatory signals, such as those released by immune cells or tissue damage, TRPA1 has emerged as a key player in the integration of inflammatory pathways within the gastrointestinal environment. Its expression in immune cells such as macrophages positions TRPA1 as a modulator of immune responses and influences the release of pro-inflammatory mediators. The role of TRPA1 in modulating the anti-inflammatory effect of Hsp90 inhibition via 17-(allylamino)-17-demethoxygeldanamycin (17-AAG) during lipopolysaccharide (LPS) or phorbol 12-myristate 13-acetate (PMA) stimulation was investigated in RAW 264.7, a mouse macrophage cell line, and PMA-differentiated THP-1 cells, a human monocytic cell line resembling macrophages (Radhakrishnan et al., 2023). Activation of TRPA1 with AITC demonstrated an anti-inflammatory effect by enhancing Hsp90 inhibition-mediated responses to LPS or PMA stimulation in macrophages. Conversely, antagonizing TRPA1 with 1,2,3,6-Tetrahydro-1,3-dimethyl-N-[4-(1-methylethyl) phenyl]-2,6-dioxo-7 H-purine-7-acetamide,2-(1,3-Dimethyl-2,6-dioxo-1,2,3,6-tetrahydro-7 H-purin-7-yl)-N-(4-isopropylphenyl) acetamide (HC-030031) downregulated these effects (Radhakrishnan et al., 2023). Macrophage activation induced by LPS or PMA is regulated by TRPA1. Additionally, TRPA1 has been identified as a significant contributor to intracellular calcium levels during Hsp90 inhibition in LPS- or PMA-stimulated macrophages (Radhakrishnan et al., 2023).

The involvement of TRPA1 extends to the perception of visceral pain during inflammation (Lapointe and Altier, 2011). Sensitization of TRPA1 in sensory nerve fibers enhances pain perception, contributing to the visceral hypersensitivity observed in inflammatory conditions. This nociceptive role underscores the participation of TRPA1 in the complex interplay between inflammation and pain perception in the gastrointestinal tract (Lapointe and Altier, 2011). Notably, in the colon, mechanical hypersensitivity induced by TRPA1 agonists increased in afferents of mice with chemically induced colitis. This implies a potential role of TRPA1 in mechanosensory function and sensitization under inflammatory conditions (Brierley et al., 2009). TRPA1 contributes to pancreatic pain, and TRPA1 also mediates pancreatic inflammation (Schwartz et al., 2011). TRPA1 is expressed in visceral afferent neurons and participates in inflammatory responses and the establishment of hypersensitivity (Ceppa et al., 2010). Within an intricate network of enteric neurons, TRPA1 exerts a significant effect on gut motility (Ye et al., 2021). Its activation in enteric neurons contributes to the regulation of peristalsis and the overall gastrointestinal transit. TRPA1-mediated signals play a crucial role in coordinating smooth muscle contractions and ensuring the propulsive movements necessary for effective digestion and nutrient absorption (Cho et al., 2014). TRPA1 is present in enterochromaffin (EC) cells within the enteric mucosa, displaying a graded expression profile along the length of the gut and is notably absent from the colon (Cho et al., 2014; Hassan et al., 2020).

**FIGURE 4:**
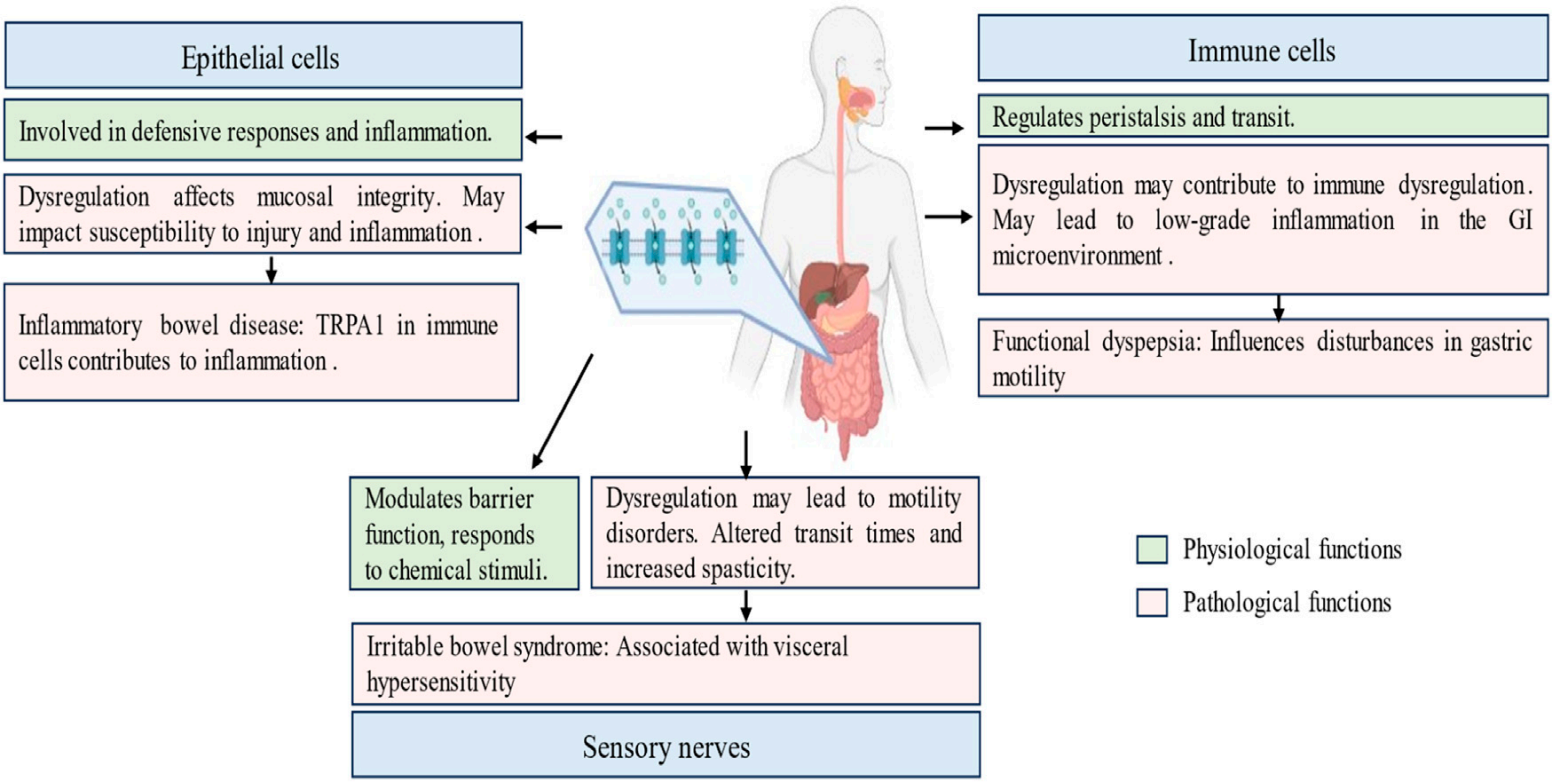
Physiological and pathological roles of transient receptor potential ankyrin 1 (TRPA1) channel in the gastrointestinal tract. This figure navigates the pivotal role played by TRPA1 within the gastrointestinal tract, elucidating its involvement in sensory perception, immune modulation, and neuropsychiatric regulation. It explores the significance of TRPA1 in conditions such as functional dyspepsia, inflammatory bowel disease and irritable bowel syndrome. It highlights the presence of TRPA1 in epithelial cells, immune cells and enteric neurons, emphasizing its diverse contribution to gut motility, mucosal defense, and nociceptive signaling.

TRPA1 activation influences the balance between muscle contraction and relaxation. Dysregulation of TRPA1 signaling may contribute to motility disorders, affecting the overall efficiency of the gastrointestinal propulsion process (Linan-Rico et al., 2016; Yang et al., 2019). Agonists and antagonists targeting TRPA1 have demonstrated effectiveness in treating neuropsychiatric disorders and appetite regulation, establishing a vital connection between the two. TRPA1 channels play a role in regulating appetite, lipid metabolism, glucose and insulin homeostasis, and the inflammation associated with neuropsychiatric and metabolic disorders (Sodhi et al., 2021). Non-electrophilic TRPA1 activators, including menthol, carvacrol, and clotrimazole, induce an increase in the permeability of fluorescein isothiocyanate-conjugated dextran (4 kDa) and a decrease in transepithelial electrical resistance in epithelial MDCK II monolayers. This effect is accompanied by Ca2+ influx and cofilin activation (Mukaiyama et al., 2020). Pretreatment with a TRPA1 antagonist attenuated these phenotypes, suggesting a TRPA1-mediated opening of tight junctions. These findings imply that nonelectrophilic TRPA1 activators, known for their safety, can be employed to modulate epithelial barriers (Mukaiyama et al., 2020). The involvement of TRPA1 in visceral pain perception extends beyond inflammation and includes broader nociceptive pathways. The role of TRPA1 in the gastrointestinal tract, disorders, cell types, and functions are detailed in Figure 4.

### 5.3 Implications in gastrointestinal disorders

The TRPA1 channel, a versatile sensory receptor in the gastrointestinal (GI) tract, has emerged as a pivotal player in the pathophysiology of various GI disorders (Brierley et al., 2009). This extensive exploration delves into the profound and complex links between TRPA1 dysregulation and gastrointestinal maladies, including Irritable Bowel Syndrome (IBS) (Choi et al., 2023), Inflammatory Bowel Disease (IBD) (Cseko et al., 2019), and Functional Dyspepsia (Balemans et al., 2017), comprehensively illustrating how aberrant TRPA1 signaling contributes to the complexity of these disorders. A hallmark feature of IBS is visceral hypersensitivity, in which the perception of normal GI stimuli is amplified, leading to abdominal pain and discomfort. TRPA1, which is abundantly expressed in sensory nerves throughout the GI tract, plays a pivotal role in transducing noxious stimuli (Kaji et al., 2012). TRPA1 dysregulation may contribute to visceral hypersensitivity, heightened pain perception, and exacerbated IBS symptomatology (Balemans et al., 2017). Visceral hypersensitivity was less prevalent in elderly patients with IBS. Healthy elderly individuals show a significant decrease in the expression of TRPA1, indicating its potential involvement in the modification of visceroperception (Beckers et al., 2021). Low-grade inflammation and immune dysregulation have been observed in a subset of patients with IBS. TRPA1 is expressed not only in sensory nerves but also in immune cells and may contribute to immune modulation within the GI microenvironment (Eissmann et al., 2020; Naert et al., 2021). Dysregulated TRPA1 activation in immune cells may perpetuate low-grade inflammation and contribute to chronic IBS symptoms (Burns et al., 2022).

Inflammatory Bowel Disease (IBD) encompasses Crohn’s disease and ulcerative colitis and is characterized by chronic relapsing inflammation of the GI tract (Coates and Binion, 2021). TRPA1 expression in immune cells places it at the crossroads of immune-mediated inflammation. Dysregulation of TRPA1 in immune cells may contribute to the release of pro-inflammatory mediators, thereby influencing the perpetuation of inflammation observed in IBD. Despite the inconsistencies in findings over the last 2 decades, preclinical evidence and limited human studies have indicated the potential therapeutic efficacy of TRPV1 and TRPA1 antagonists in treating colitis and visceral hypersensitivity. These channels present a distinct mechanism of action for drug development in the context of inflammatory bowel disease (IBD) (Cseko et al., 2019). Visceral pain experienced by individuals with IBD is multifactorial, and the role of TRPA1 in pain perception is crucial. Dysregulated TRPA1 signaling in sensory nerves may amplify pain responses, contributing to the heightened visceral pain experienced during IBD flares. This nociceptive amplification may significantly affect the quality of life of patients with IBD (Chen et al., 2020).

Mice lacking TRPA1 displayed reduced dermal thickening, diminished collagen accumulation, and decreased expression of pro-fibrotic factors. Blocking TRPA1 potentially relieves conditions such as systemic sclerosis and scleroderma (Maki-Opas et al., 2023). IBD is often associated with disruption of mucosal integrity. TRPA1, which is involved in the mucosal defense mechanisms, may contribute to the delicate balance between protection and damage. Dysregulation of TRPA1 signaling might influence mucosal defense mechanisms, potentially affecting the susceptibility of the GI mucosa to injury and inflammation. TRPA1 is implicated in abdominal pain and hypersensitivity in irritable bowel syndrome (IBS) and Inflammatory Bowel Disease (IBD). One study underscored the potential influence of capsazepine treatment on pain and mucosal health via TRPA1/TRPV1 modulation (Kumar et al., 2022). However, the observed side effects such as mucous layer loss necessitate cautious consideration of their impact, whether detrimental or adaptive. Before clinical application, a deeper understanding of the role of nociceptors in mucin health is crucial for refining therapeutic approaches (Kumar et al., 2022). Functional dyspepsia is a gastrointestinal disorder of gastroduodenal origin within the category of functional GI disorders and is characterized by symptoms such as postprandial fullness and early satiety; it often involves disturbances in gastric motility, including gastric peristalsis, and is a potential contributor to the dysmotility patterns observed in functional dyspepsia (Sayuk and Gyawali, 2020). Dysregulated TRPA1 signaling may influence the rhythmic contractions necessary for effective digestion.

### 5.4 Cross-cutting themes

Psychological factors play a significant role in causing gastrointestinal disorders (Wilhelmsen, 2000). Stress and anxiety can modulate TRPA1 activity, potentially exacerbating symptoms in individuals with TRPA1 dysregulation and contributing to the complex interplay between the gut and brain (Hassan et al., 2020; Giacco et al., 2023; Sullivan et al., 2023). Understanding the link between TRPA1 dysregulation and GI disorders opens new avenues for targeted therapeutic interventions. The modulating of TRPA1 activity, either through pharmacological agents or lifestyle modifications, presents a potential strategy for alleviating symptoms and improving the quality of life of individuals with complex and heterogeneous disorders. The potential links between TRPA1 dysregulation and GI disorders such as IBS and IBD unravel a complex web of interactions within the GI milieu (Chen et al., 2020; Lin et al., 2021). TRPA1 has emerged as a central player in orchestrating GI homeostasis (Lapointe and Altier, 2011; Yu et al., 2016; Gong et al., 2023). Unravelling the intricacies of TRPA1 dysregulation offers a tantalizing glimpse into the molecular underpinnings of these disorders and presents opportunities for innovative and targeted therapeutic interventions in the field of gastroenterology.

## 6 Interactions and cross-talk

### 6.1 Integration of respiratory and gastrointestinal TRPA1 signaling

The TRPA1 channel, a versatile sensory receptor, orchestrates a symphony of responses in both the respiratory (Jha et al., 2015) and gastrointestinal (Yang et al., 2019) tracts. Within the respiratory tract, the strategic positioning of TRPA1 in epithelial cells lining the airways makes it a frontline sensor of inhaled irritants and environmental challenges (Omar et al., 2017). Its activation initiates immediate protective responses, preserving airway integrity and triggering reflexes such as coughing and bronchoconstriction (Grace and Belvisi, 2011; Al-Shamlan and El-Hashim, 2019; Jentsch Matias de Oliveira et al., 2020). The role of TRPA1 extends beyond the epithelium to sensory nerves and immune cells, contributing to nociception, immune responses, and inflammatory processes (Fischer et al., 2008; Luostarinen et al., 2023; Muthumalage and Rahman, 2023). Dysregulation of TRPA1 in conditions such as severe asthma and fibrosis underscores its significance in airway remodeling and respiratory homeostasis (Yang and Li, 2016; Li et al., 2020; Yap et al., 2021; Li et al., 2022).

In the cross-talk between the respiratory and gastrointestinal tracts, the involvement of TRPA1 in immune cells is evident (Billeter et al., 2014; Naert et al., 2021). In the respiratory microenvironment, TRPA1 is expressed in macrophages and mast cells and influences immune responses and inflammatory processes (Naert et al., 2021). This immune modulation by TRPA1 may extend to the gastrointestinal tract, where immune cells, including macrophages, express TRPA1 (Chen et al., 2020). The shared presence of TRPA1 in immune cells suggests a potential interplay between the regulation of inflammatory responses in both systems.

In the gastrointestinal tract, TRPA1 exhibits a dynamic expression pattern in various segments, including enteric neurons (Cho et al., 2014; Hassan et al., 2020), epithelial cells (Kono et al., 2013), and immune cells (Naert et al., 2021). Activation of TRPA1 in gut sensory neurons influences peristalsis and sensitivity to mechanical and chemical stimuli, and plays a crucial role in gastrointestinal motility (Tsuchiya and Kawamata, 2019). The parallel role of TRPA1 in airway reflexes and sensitivity in the respiratory tract suggests a common mechanism for orchestrating protective responses and reflex arcs in both systems (Mukhopadhyay et al., 2016).

Moreover, the participation of TRPA1 in the perception of pain was evident in both the respiratory and gastrointestinal tracts. In the respiratory system, TRPA1 activation in sensory nerves leads to a cough reflex and bronchoconstriction (Grace and Belvisi, 2011; Jentsch Matias de Oliveira et al., 2020), whereas in the gastrointestinal tract, TRPA1 sensitization in sensory nerve fibers contributes to visceral hypersensitivity and abdominal pain (Beckers et al., 2021; Landini et al., 2022). The interconnection between TRPA1-mediated pain responses in both the systems is a shared mechanism in the modulation of sensory perception.

The role of TRPA1 in inflammation is another point of intersection. In the respiratory tract, TRPA1 is implicated in airway tissue injury and inflammation (Wang M. et al., 2019), whereas in the gastrointestinal tract, dysregulated TRPA1 activity in gut sensory neurons may contribute to inflammation observed in conditions such as irritable bowel syndrome (IBS) (Landini et al., 2022) and inflammatory bowel disease (IBD) (Kumar et al., 2022). This shared involvement in inflammatory processes highlights the potential of TRPA1 as a therapeutic target for conditions involving immune dysregulation in both systems.

The cross-talk between the respiratory and gastrointestinal tracts with respect to TRPA1 reveals commonalities in its spatial dynamics, protective responses, immune modulation, pain perception, and regulation of inflammation. Understanding the versatile role of TRPA1 in orchestrating these responses across different cell types in both systems lays the foundation for targeted therapeutic interventions in respiratory and gastrointestinal conditions where TRPA1 dysregulation plays a pivotal role.

TRPA1 channels in the respiratory and gastrointestinal tract reveals limitations in understanding the precise role of these channels in disease pathogenesis. While this review may offer insights into potential therapeutic targets for respiratory and gastrointestinal conditions, it is constrained by the complexity of cellular and molecular interactions within these systems. Additionally, the findings may not fully capture the dynamic nature of TRPA1 channel activity in various physiological and pathological contexts. Moreover, changing these findings into clinical applications may be complicated by differences in experimental models and human physiology. Further research is needed to elucidate the specific mechanisms underlying TRPA1 channel function and its implications for respiratory and gastrointestinal health.

## 7 Conclusion and areas for future research

The exploration of the TRPA1 in both the respiratory and gastrointestinal tracts has revealed its multifaceted role as a pivotal molecular orchestrator. Within the respiratory system, TRPA1 is present in the epithelial cells, sensory nerves, and immune cells, thereby serving as a frontline sensor for inhaled irritants and other environmental challenges. Its activation initiates immediate protective responses, regulates long-term physiological processes, and affects immune responses. Dysregulation of TRPA1 in conditions such as asthma and chronic obstructive pulmonary disease (COPD) underscores its significance in respiratory homeostasis and presents opportunities for targeted therapeutic interventions.

Furthermore, in the gastrointestinal tract, the dynamic expression pattern of TRPA1 in enteric neurons, epithelial cells, and immune cells positions it as a central player in orchestrating responses to a myriad stimuli, ranging from dietary components to inflammatory signals. TRPA1 influences gut motility, visceral pain perception, and mucosal defense mechanisms and plays a crucial role in maintaining gastrointestinal homeostasis. Dysregulation of TRPA1 been implicated in various gastrointestinal disorders including irritable bowel syndrome (IBS), inflammatory bowel disease (IBD), and functional dyspepsia, contributing to visceral hypersensitivity, inflammation, and disturbances in gastric motility.

The therapeutic implications of targeting TRPA1 in respiratory and gastrointestinal disorders are promising. The development of TRPA1 antagonists, such as BI01305834 and GDC-0334, has demonstrated efficacy in preventing airway hyperresponsiveness, bronchoconstriction, and abnormal colorectal contractions. These findings offer hope for the development novel treatment strategies for asthma, COPD, IBS, and IBD. However, the complex interplay of TRPA1 in immune modulation and nociceptive pathways requires further research to unravel its detailed functional properties in specific cell types and signaling pathways.

This comprehensive review underscores the versatile and crucial role played by TRPA1 in chemical sensing, sensory transduction, and immune modulation in both the respiratory and gastrointestinal systems. The spatial nuances of TRPA1 across different cell types revealed a cellular symphony that influences immediate protective responses, long-term physiological processes, and inflammatory conditions. Understanding TRPA1 in these contexts lays the foundation for targeted therapeutic interventions and paves the way for future research aimed at unravelling the intricacies of its involvement in health and diseases.

The systemic impact of TRPA1 dysregulation along with how conditions in one system influence another, particularly in the context of systemic inflammation, were explored. Additionally, the influence of psychosomatic factors on TRPA1 activity as well as the impact of stress and anxiety on physiological responses were further investigated. Development and exploration of therapeutic interventions targeting TRPA1 for dual-system applications, considering conditions in which both the respiratory and gastrointestinal systems are implicated.

TRPA1 is a versatile molecular conductor in both the respiratory and gastrointestinal tracts that influences sensory perception, inflammatory responses, and physiological homeostasis. The shared expression patterns and functional roles highlight the interconnectedness between these systems. Unravelling the complex nuances of TRPA1 in both domains not only deepens our understanding of physiological regulation but also opens avenues for innovative therapeutic approaches that transcend traditional organ-specific boundaries. Future research should explore the systemic impact of TRPA1 dysregulation, psychosomatic influences, and the potential of dual-system therapeutics to address conditions in which both systems play a role.
